# Increasing Confidence of Proteomics Data Regarding the Identification of Stress-Responsive Proteins in Crop Plants

**DOI:** 10.3389/fpls.2016.00702

**Published:** 2016-05-24

**Authors:** Xiaolin Wu, Wei Wang

**Affiliations:** State Key Laboratory of Wheat and Maize Crop Science, Collaborative Innovation Center of Henan Grain Crops, College of Life Science, Henan Agricultural UniversityZhengzhou, China

**Keywords:** crop plants, abiotic stress, stress-responsive proteins, proteomics data, 2DE, iTRAQ labeling

## Introduction

Numerous stresses caused by complex environmental conditions, e.g., drought, heat, cold, salinity, strong light, UV, and heavy metals, negatively affect plant growth and lead to substantial crop losses worldwide. It is estimated that up to 50–70% of declines in crop productivity can be attributed to abiotic stress (Mittler, 2006). Abiotic stress, particularly drought and extreme temperatures, will be more frequent and severe in the near future because of global climate change (Horton et al., 2015). Understanding the abiotic stress response in plants has attracted substantial attention within the plant proteomics community.

Quantitative proteomic comparisons are particularly useful in defining proteins that change in abundance, form, location, activity, and these comparisons may indicate involvement in responses to alterations in environmental conditions (Thelen and Peck, 2007). Such analyses can detect proteins involved in the mechanisms underlying plant stress resistance to various abiotic stresses. These proteins can potentially serve as molecular markers in marker-assisted selection by possibly speeding up the identification of relevant targets for stress breeding.

Considering the 2DE and/or iTRAQ analysis methods of proteomics as an example, we briefly analyzed the methodological defects in detecting stress-responsive proteins in plants and propose our opinions for addressing these defects in future plant stress proteomics. The intended audiences of this opinion paper are novice rather than experienced scientists in the plant proteomics research community.

## Methodological defects in plant stress proteomics

Comparative proteomics detection of stress-responsive proteins in plants is performed through analyzing protein changes, including protein isoforms and molecular species generated by PTMs, between untreated and stress-treated samples or tolerant and intolerant plants. An increasing number of studies indicate that protein changes are important in plant stress response (e.g., reviews by Kosová et al., 2011; Barkla et al., 2013; Ghosh and Xu, 2014; Wu et al., 2016). Based on briefly reading the abstracts of these studies, it is obvious that 2DE-based approaches and iTRAQ-based approaches currently represent two major types of proteomics techniques in plant stress proteomics.

The 2DE method resolves proteins based on a native charge followed by mass (Rabilloud et al., 2010). The routine 2DE approach allows the detection of lower numbers of protein spots (compared to iTRAQ), and subsequent mass spectrometry-based identification can be applied only to differentially abundant stress-responsive proteins among the analyzed samples. Moreover, 2DE appears to be especially suitable for the detection of changes on the level of protein isoforms (Benešová et al., 2012). A disadvantage of 2DE is that spot matching among a group of 2DE gels can be an arduous task (Thelen and Peck, 2007; Rabilloud et al., 2010). Though DIGE-2DE can make comparisons easier, high-quality 2DE gels with minimal spot streaking and overlap are critical to simplify and maximize the accuracy of spot matching. In good instances, impressive 2DE maps seem like exquisite artistic works compared to the masses of lifeless iTRAQ data.

The iTRAQ analysis method is a second-generation proteomic technique that provides a gel-free shotgun quantitative analysis. It utilizes isobaric reagents to label tryptic peptides and monitor relative changes in protein and PTM abundance (Ross et al., 2004), and it allows for the comparison of up to eight samples. Thus, iTRAQ especially facilitates the analysis of time courses of plant stress responses or biological replicates in a single experiment. However, iTRAQ monitors several thousands of peptides without the ability to pre-select differentially abundant peptides prior to mass spectrometric identification. Compared to 2DE, iTRAQ requires intensive data analysis using appropriate software to detect and quantify the mass tags. To our knowledge, many iTRAQ analyses had been performed by commercial services. Quite often, it works like a “black box.” The customers submit samples and get a list of differential proteins with ratios, without knowing the details of experimental processes. During the commercial iTRAQ analysis, experimental design may not be well taken care of, protein isolation may not be properly conducted, and experimental data may not be properly analyzed, which all contribute toward the distortion of iTRAQ data.

Comparative proteomics studies provide a great deal of data and novel insights on plant stress response. However, substantially inconsistent or unreliable results occur in plant stress proteomics research among different research groups. For example, this inconsistency is clear in the comparison of proteomic studies of maize (*Zea mays*) under salt and drought stresses using 2DE or iTRAQ approaches (Table 1).

**Table 1:** **Examples of detection of stress-responsive proteins in maize by 2DE and/or iTRAQ proteomic approaches**.

**Maize**	**Stress treatment**	**Organ**	**Protein extraction and dissolving**	**Differential detection**	**Differential proteins**	**Common differential proteins**	**References**
**SALT STRESS**							
Hybrid line SR12	25 mM NaCl for 1 h, 20-d-old seedlings	Root	TCA/Acetone precipitation Proteins dissolved in 8 M urea, 2 M thiourea, 4% CHAPS, 30 mM DTT, 2% pharmalyte buffer, 20 mM Tris, 5 mM Pefabloc, etc.	2DE, pH 3–10 IPG strip CBB staining MALDI-TOF MS	29	Three, i.e., fructokinase-2, sucrose synthase, and 14-3-3-like protein.	Zörb et al., 2010
Genotypes N1145 (tolerant) D340 (sensitive)	150 mM NaCl for 3 d at 3-leaf stage	Root	Phenol extraction Proteins dissolved in 7 M urea, 2 M thiourea, 4% CHAPS, 1% DTT, 0.8% IPG buffer	2DE, pH 4–7 strip IPG Silver staining MALDI-TOF/TOF MS	57		Cheng et al., 2014
Genotypes F63 (tolerant) F35 (sensitive)	160 mM NaCl for 2 d at 3-leaf stage	Root	TCA/Acetone precipitation Proteins dissolved in 6 M urea, 2% CHAPS, 50 mM trethylammonium bicarbonate	iTRAQ LC-MS/ MS	28		Cui et al., 2015
**DROUGHT STRESS**							
Genotypes CE704 (tolerant) 2023 (sensitive)	Withhold water for 6 d at 4-leaf stage	Leaf	TCA/Acetone precipitation Proteins dissolved in 8 M urea, 0.5–4% CHAPS and 0.4% DTT	2DE, pH 4–7 IPG strip Silver staining MALDI-TOF MS/MS iTRAQ, LC-MS/MS	11 (2DE) 220 (iTRAQ)	Four, i.e., phosphoglucomutase peptidyl-prolyl cis-trans isomerase, GST1, and UDP-glucosyltransferase BX9.	Benešová et al., 2012
Inbred line KS140	Withhold water for 10 d at 6-leaf stage	Leaf	Phenol extraction Proteins dissolved in 9.5 M urea, 4.5% acrylamide, 2% NP-40, 2.5% pharmalytes	2DE, pH 4–7 IPG strip CBB staining MALDI-TOF MS	29		Kim et al., 2015

In the three studies on proteomic changes in maize roots under salt stress, only three identified proteins were common among the dozens of differentially abundant proteins; only four proteins were common in two proteomic studies of maize leaf under drought conditions (Table 1). Even in a single experiment, only four stress-responsive proteins were identified by both the 2DE and iTRAQ analyses (Benešová et al., 2012). The partial overlap between the outputs of 2DE and iTRAQ approaches was limited due to their different characteristics (Alvarez et al., 2009; Benešová et al., 2012). Only relatively abundant proteins within a limited *p*I range (e.g., 3–10) can be detected by standard 2DE, whereas the iTRAQ method allows for the analysis of proteins present in low quantities and ones that tend to be difficult to separate by 2DE. However, a key question is that many abundant proteins detected by 2DE cannot be detected by iTRAQ. This is probably due to the lack of distinction of protein isoforms when the ratios are quantified by iTRAQ. Most of current software for iTRAQ (except for ProteinPilot) cannot discriminate abundance changes of different isoforms; therefore, if a protein abundance change resulted from the increase/decrease of a certain isoform, or the presence/absence of a certain PTM(s), the iTRAQ ratio may not show significant change. It is obvious that iTRAQ has also considerable deficits in differential protein detection.

Currently, the identification of stress-responsive proteins in crop plants is poorly overlap among different groups, even using state-of-the-art instrumentation. Except for differences in plant genotype, growth, and stress conditions, the inconsistent or unreliable results regarding identification of stress-responsive proteins mainly originate from erroneous methodology. In particular, three prominent problems affect the accuracy and reliability of proteomic data.

The first problem is inappropriate protein-extraction methods for plant tissues. Compared to model plant *Arabidopsis*, crop plants are more problematic in protein extraction, because they contains large amounts of secondary compounds such as phenolics, lipids, and organic acids, which severely interfere with protein extraction and proteome analysis (Wu et al., 2014a). Generally, protein-extraction methods need to be optimized and improved dependent on plant species and tissue types. Due to the great variance in sets of secondary metabolites present in various tissues from diverse plant species, no single extraction protocol is effective for every tissue. Adult tissue is usually more problematic than young tissue. For a given tissue, it is recommended that protein extraction starts with simple TCA/acetone precipitation and/or phenol extraction, and is then modified accordingly. Previously, we introduced some cases of protein extraction methods from representative plant tissues for proteomic analysis (Wang et al., 2008). The specific methods used in different labs will cause the disagreement in proteomic results, even for the same tissue and/or treatment.

The second problem is the poor quality of the original proteomics data, especially 2D gels, which is usually due to methodological issues during protein extraction and analysis, e.g., incomplete extraction, interference of non-protein substances, incomplete focusing, and incorrect spot matching. Generally, 2DE is performed manually. Poor-quality 2DE maps will result in erroneous or inconsistent results. Although these common problems intrinsic to 2DE are well known to the proteomics research field, novices in the plant proteomics community should make efforts to improve protein extraction and analysis, dependent on specific experiments, and crop species.

The third problem is insufficient replicates in 2DE or iTRAQ analysis. Many studies using 2DE or iTRAQ analysis have claimed to include three or more independent biological replicates, but these studies did not provide the relevant figures or data. Alvarez et al. (2009) indicated that the quality of iTRAQ results depends on both the number of biological replicates and the number of sample injections. In iTRAQ analysis, despite the application of quality assurance protocols, most errors occur during the pre- and analytical phases. Commercial iTRAQ services quote a price of approximately $5000–10,000 for a single iTRAQ analysis of four to eight samples. It is conceivable that iTRAQ analysis sometimes lacks sufficient and necessary biological or technical replicates due to expense. Another possibility is the consequence of the pressure exerted by the well-known “publish or perish” dilemma, which often results in the rapid and careless publication of data (Fernández-Marín et al., 2015).

In some instances, differential protein changes in abundance were not as significant as reported. Kim et al. (2015) detected 29 differentially abundant spots in maize leaves under drought stress. However, upon comparing the relative abundance of the differentially expressed proteins, we found that only 10 proteins showed 1.5–2.0-fold changes in abundance, whereas the other seven proteins showed no obvious changes in abundance. Therefore, authors should perform more careful and thorough checking of stress-induced differentially abundant proteins before publication. The novice in plant proteomics also should pay attention to the articles published in the journals by those so-call predatory publishers (https://scholarlyoa.com/publishers/).

## Concluding remarks

As discussed above, due to the weakness in the quality of proteomic data and the constraints on biological and technical replicates, it is not surprising that few commonalities and limited biological significance can be drawn from the numerous studies from different groups regarding plant stress proteomics.

To improve the accuracy of detection of stress-responsive proteins in plants, novices in the plant proteomics community must give higher consideration to sample preparation prior to gel-based or gel-free proteomics analysis. The quality of 2DE maps is very straightforward, so many protein extraction protocols have been reported based on a 2DE evaluation, whereas almost none have been reported for protein extraction protocol evaluation by iTRAQ analysis. Recently, we reported in detail a universal protein extraction protocol integrating TCA/acetone precipitation with phenol extraction (Wu et al., 2014b). This protocol made it possible to obtain satisfactory 2DE maps of various crop plant tissues, and it could be suitable for gel-free approaches. In addition, organelle isolation and/or protein fractionation techniques during sample preparation can improve the depth of proteome analysis through reducing proteome complexity.

Another aspect to consider for improving the accuracy of detection of stress-responsive proteins in plants is that the publication of proteomic data should describe biological and technical replicates and provide the necessary proteomic data on the replicates. As proteomics (especially the quantitative approach) is a statistically based method that relies on probability and arbitrary thresholds, there is always the chance of reporting false positives. To obtain proteomic data of confidence, the biological replicates should be at least three times, with three technical replicates in an independent biological experiment.

Moreover, proteomic experiments should be conducted within financial constraints to allow sufficient biological and technical replicates to increase the confidence of the proteomics data. When experiments are designed and performed properly, the technical variation should be comparable between methods, and the results show good agreement and biological significance.

Finally, experimental validation is often required to increase the confidence of the proteomic results, which can be carried out by qRT-PCR or transcriptomic analysis, or can be verified by using specific antibodies through immunochemistry, or directly measuring the changes of enzyme activity.

## Author contributions

XW drafted the paper and WW edited the paper.

### Conflict of interest statement

The authors declare that the research was conducted in the absence of any commercial or financial relationships that could be construed as a potential conflict of interest.
